# Impact on urban river water quality and pollution control of water environmental management projects based on SMS-Mike21 coupled simulation

**DOI:** 10.1038/s41598-024-57201-z

**Published:** 2024-03-18

**Authors:** Huaibin Wei, Yiding Rao, Jing Liu, Yao Wang, Yongxiao Cao

**Affiliations:** 1 School of Management and Economics, North China University of Water Resources and Electric Power, Zhengzhou, 450046 Henan China; 2 School of Water Conservancy, North China University of Water Resources and Electric Power, Zhengzhou, 450046 Henan China; 3 School of Water Resources, North China University of Water Resources and Electric Power, Zhengzhou, 450046 Henan China; 4 The Key Laboratory of Conservation and Intensive Utilization of Water Resources in the Yellow River Basin of Henan Province, Zhengzhou, 450046 Henan China

**Keywords:** Hydrodynamic-water quality coupling simulation, SMS-Mike21 coupling model, Hydrology period, Water environmental management projects, Urban river, Environmental sciences, Hydrology

## Abstract

To explore the impact of expanding Nanyang Sewage Purification Center (NSPC) on the main sewage discharge area of Bai River, we constructed a 2D hydrodynamic-water quality model based on surface water modeling system (SMS) and Mike21. Simulating three sewage discharge conditions in wet, normal, and dry season, we evaluated three indicators (COD, NH_3_-N, and BOD_5_) by the single-factor pollution index and provided recommendations for water environment management. The results showed that, maximum absolute error of water level was 0.08 m, percentage bias coefficient of COD, NH_3_-N and BOD_5_ were 19.3%, 16.2% and 23.1%, indicating the SMS and Mike21 coupling model was applicable; water quality of the assessment section were upgraded from the original class IV, V, V (Condition 1) to class IV, III, II (Condition 2) and class IV, III, III (Condition 3) in the wet, normal and dry season, indicating that NSPC's expansion had improved the water quality of the assessment section; as the primary pollutant, BOD_5_ concentration in the downstream was lower than the upstream, which was due to the dilution effect of river. Therefore, on the basis of expanding NSPC, we recommend to remediation of BOD_5_ by physical, chemical, and biological methods. This study broadens new ideas for the application of Mike21, and provide a reference for the prevention and improvement of river water pollution in urban areas.

## Introduction

As a population and industrial agglomeration area, population expansion and industrial development had increased the amount of sewage into urban rivers, which had hindered the development of cities^1,2^. When studying the changes of the rivers’ water environment, the hydrodynamic-water quality model is often used to simulate the water environment, and then figure out the water environment problems and put forward targeted measures, which has strong practical significance and guiding significance for water environment governance^3–6^.

Hydrodynamics and water quality are the two characteristics of water environment. Using real measured data, so as to investigate the influence of hydraulic conditions on water quality, and then construct a coupling model of hydrodynamic-water quality to predict water environment, it is a key direction of international water environment research^7–9^. The hydrodynamic-water quality model can not only quantitatively describe the water environment system and the complex processes that occur within it, but also intuitively display the scope and degree of pollution impact on the layer of geographic information system, so as to provide decision-making basis for the early warning and prevention of water pollution accidents^10,11^. During the years of practice, the hydrodynamic-water quality model has developed zero-dimensional model, 1D model, 2D model, 3D model and different dimensional model coupling^9–12^. Different kinds of model have different simulation priorities and applicability. The zero-dimensional model does not contain an input–output model of hydrodynamic information, only assumes the water body is well mixed. The 1D model can simplify the complex river network, and is suitable for the situation with little change in the horizontal and vertical directions, which is advantageous of high computational efficiency^13^. The computational result of the 2D model is more detailed, which can accurately simulate local flow patterns and pollutant migration, and is suitable for wider rivers and lakes^14^. The 3D model comprehensively considers the hydrodynamics and water quality changes of the three dimensions, and the calculation results are more accurate and comprehensive, but the calculation is a huge quantity^15^. The different dimensional model coupling is often constructed according to the characteristics of the study object and study purpose, then the model’s applicability is stronger. For example, the MIKE-11 model and the MIKE-21 model were coupled to simulate the flood inundation of the Ahoi River^16^. In this paper, the river’s channel is wide, the 2D hydrodynamic-water quality coupling model is applicable. While Mike21 has a multi-functional module and powerful pre-and post-processing functions, which can better reflect the influence of river bottom fluctuations on water level, flow regime and water quality, and is widely used in hydrodynamic-water quality simulation of rivers, lakes and reservoirs^17–19^. At the same time, considering that the mesh generated by SMS has more advantages in the computational speed and the convergence speed of the solution, we use SMS and Mike21 to establish a 2D hydrodynamic-water quality model, which is conducive to improve the model’s accuracy^20^. In addition, in the past studies on the hydrodynamic-water quality models, few scholars considered the water quality in different hydrology periods^21,22^, when setting the sewage discharge scenario, we comprehensively consider the upstream water volume and quality of the studied river in different hydrology periods, which can make the model closer to the actual situation and provide a scientific theoretical basis to refine the water pollution control of the river.

According to the water quality evaluation from 2017 to 2021 by the water environment quality monitoring network of Henan Province, it showed that the water quality indicators COD, NH_3_-N and BOD_5_ of the main sewage discharge area of the Bai River (Nanyang section) continuously exceed the target water quality category of the assessment section. In this paper, we took the main sewage discharge area as the study area, it includes all the sewage discharge outlets in the Bai River (Nanyang section), and its downstream section is designated as a water quality control section by the water administrative authority. So we used SMS and Mike21 to establish a 2D hydrodynamic-water quality coupling model for it, and selected COD, NH_3_-N, and BOD_5_ as water quality indicators, and designed nine sewage discharge scenarios from the perspectives of hydrology periods and sewage discharge into the river, the hydrology periods was divided into wet, normal and dry season, and the sewage discharge into the river is divided into Condition 1 (C1), Condition 2 (C2) and Condition 3 (C3) depending on the difference of the sewage discharge volume of each sewage discharge outlet before and after the expansion of NSPC. Based on the simulation results, we determined whether the water quality category of the assessment section meets the class III by the single factor pollution index of COD, NH_3_-N, and BOD_5_, and analyzed the spatial distribution of river water environmental pollution factors, then discussed the impact of the expansion of NSPC on the water environment of the Bai River, and proposed suggestions for the prevention and control of water environment pollution, so as to provide the scientific basis for the formulation of water environment treatment policies in the Bai River.

## Materials and methods

### Study area

The urban area of Nanyang City is located in the south of Henan Province (112°17′-112°50′ E, 32°37′–33°17′ N). Recently, with the rapid development of industrialization and urbanization, the volume of domestic and industrial wastewater drained into the urban river of Nanyang City had increased. Md Anawar et al., summarized the common measures of improving the rivers’ water environment are categorized into physical (mechanical aeration processes, water transfer or diversion and dilution, mechanical algae removal), chemical (flocculation, precipitation, oxidation, algaecides), biological (microbial bioremediation, biofilms, membrane bioreactor technology, plant purification treatment), ecological (ecological floating beds, artificial floating islands, constructed wetlands), engineering (building hydraulic structures, dredging river sediment) techniques, and hybrid techniques^23^. The sewage treatment works is a kind of water environmental management projects, with the function of decomposing and transforming the pollutants in sewage, which can reduce the discharge of water environmental pollutants, and effectively improve the urban water environment^24,25^. In order to prevent the deterioration of water quality and to promote the development of a safe water environment in the Bai River, NSPC planned to expand. NSPC is located on the north bank of the main sewage discharge area of the Bai River, with a current design scale of 200,000 m^3^/d, consisting of the first phase (100,000 m^3^/d, which was completed and put into operation in 2006) and the second phase (100,000 m^3^/d, which was completed and put into operation in 2016). With the development of urbanization and industrialization in the urban area of Nanyang City, the sewage treatment scale of NSPC cannot cover the current amount of sewage to be treated, and the municipal government plans to carry out a third phase project (200,000 m^3^/d) expansion. After the completion of the expansion, the scale of NSPC will reach 400,000 m^3^/d.

**Figure 1:**
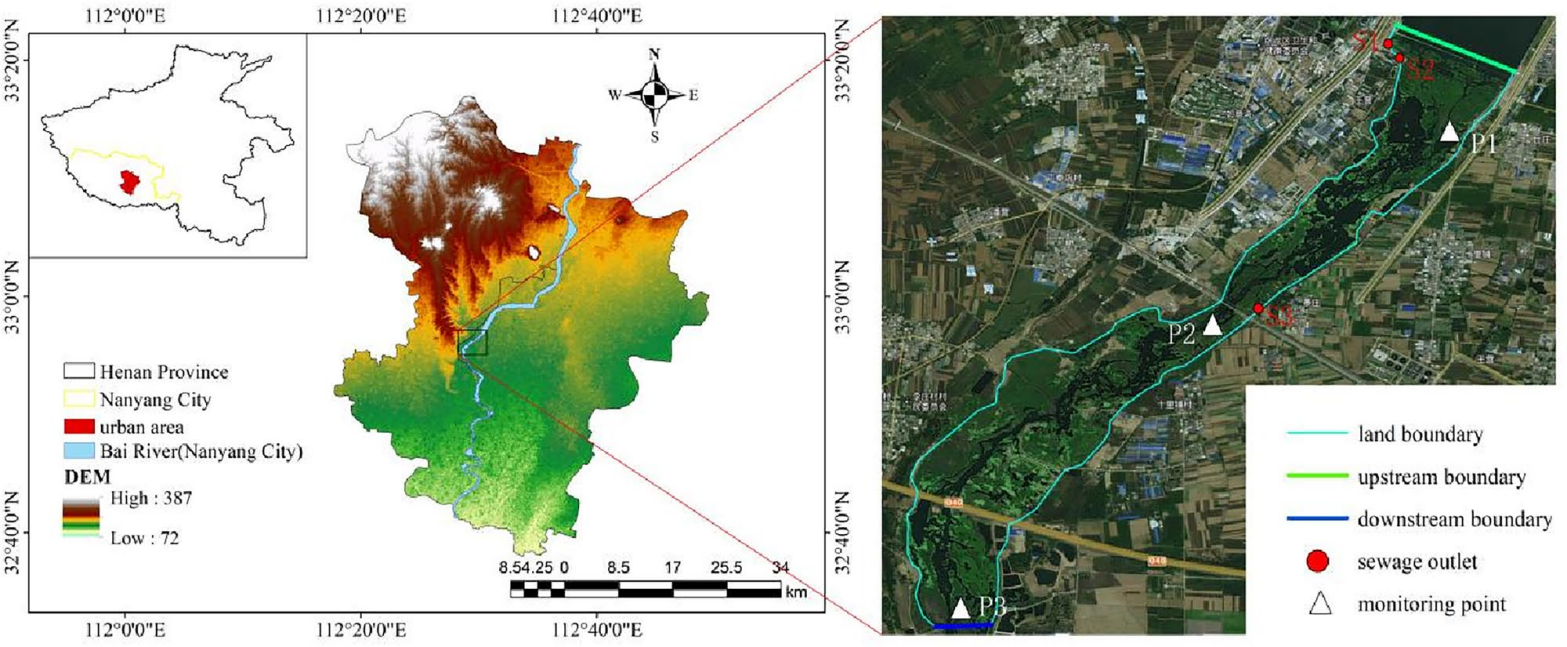
The location of the study area.

Examining the primary sewage discharge region of the Bai River (Nanyang section) as a case study, we assessed the repercussions of NSPC expansion on water quality. The Bai River, a key waterway coursing through Nanyang City’s urban expanse, is part of the primary arteries within the Han water system of the Yangtze River basin. NSPC features two sewage discharge outlets, S1 and S2, situated on the north bank of the Bai River. S1 releases untreated sewage, while S2 releases treated sewage. Additionally, on the south bank, Nanyang Tianguan Water Treatment Plant (NTWTP) has a sewage discharge outlet, S3, releasing treated sewage. Analyzing the combined impact of these three sewage discharge outlets provides a more precise understanding of their influence on the Bai River's water environment. These outlets are strategically positioned within the central area of the main sewage discharge region, as depicted in Fig. 1, which is beneficial for more comprehensive simulation of pollution discharge conditions. Figure 1 is created using ArcMap10.2, URL, www.arcgis.com. Furthermore, Fig. 1 illustrated hydrological data monitoring points, namely P1, P2, and P3. P1 served as the water level monitoring point for the fourth hydrological monitoring station, P2 as the water quality monitoring point for the Dingfengdian Section, P3 as the water quality monitoring point for the Shangfanying section. Therefore, we selected the main sewage discharge area of the Bai River (Nanyang section) as the modelling area: the upstream boundary is the Nanyang fourth rubber dam section, and the downstream boundary is the Shangfanying section, with a total length of 5 km. In this paper, the Shangfanying section is the assessment section designated by the water administrative authority, which is used to judge whether the water quality of the main sewage discharge area meets the standard or not. When the water quality category of the assessment section reaches the standard of surface water class III, it means that the water environment of the main sewage discharge area meets the standard.

### Model principle

Mike21 is a powerful 2D simulation tool for hydrodynamic-water quality of rivers, lakes, reservoirs, bays, etc. In this study, most of the main sewage discharge area of the Bai River have lateral spans of more than 200 m. Pollutant transport is characterized by obvious lateral diffusion, which cannot be uniformly mixed over a short distance. The hydrodynamic module calculates the resulting flow of forcing and boundary conditions, and the transport module computes the material transport outcome by considering the flow conditions determined in the hydrodynamic calculations. Therefore, the specific simulation process uses the Hydrodynamic Module (Mike21 HD) and the Transport Module (AD) of the Mike21 model, which is widely used to simulate water quality changes in 2D free-surface flow in wide-channel rivers.

#### Hydrodynamic model

The Hydrodynamic model (HD) adheres to the principles of three-dimensional incompressibility, Reynolds-valued homogeneous Navier–Stokes equations, and complies with the assumptions of Boussinesq and hydrostatic pressure. The Mike21 HD includes both the continuity and momentum equations^26,27^. The basic set of equations for the HD is:

1 $$ \frac{\partial h}{{\partial t}} + \frac{{\partial h\overline{u}}}{\partial x} + \frac{{\partial h\overline{v}}}{\partial y} = hS $$

2 $$ \begin{gathered} \frac{{\partial h\overline{u} }}{\partial t} + \frac{{\partial h\overline{u}^{2} }}{\partial x} + \frac{{\partial h\overline{uv} }}{\partial y} = f\overline{v} h - gh\frac{\partial \eta }{{\partial x}} - \frac{h}{{\rho_{0} }}\frac{{\partial p_{a} }}{\partial x} - \frac{{gh^{2} }}{{2\rho_{0} }}\frac{\partial \rho }{{\partial x}} + \frac{{\tau_{sx} }}{{\rho_{0} }} \hfill \\ - \frac{{\tau_{bx} }}{{\rho_{0} }} - \frac{1}{{\rho_{0} }}\left( {\frac{{\partial S_{xx} }}{\partial x} + \frac{{\partial S_{xy} }}{\partial y}} \right) + \frac{\partial }{\partial x}\left( {hT_{xx} } \right) + \frac{\partial }{\partial y}\left( {hT_{xy} } \right) + hu_{s} S \hfill \\ \end{gathered} $$

3 $$ \begin{gathered} \frac{{\partial h\overline{v} }}{\partial t} + \frac{{\partial h\overline{uv} }}{\partial x} + \frac{{\partial h\overline{v}^{2} }}{\partial y} = - f\overline{u} h - gh\frac{\partial \eta }{{\partial y}} - \frac{h}{{\rho_{0} }}\frac{{\partial p_{a} }}{\partial y} - \frac{{gh^{2} }}{{2\rho_{0} }}\frac{\partial \rho }{{\partial y}} + \hfill \\ \frac{{\tau_{sy} }}{{\rho_{0} }} - \frac{{\tau_{by} }}{{\rho_{0} }} - \frac{1}{{\rho_{0} }}\left( {\frac{{\partial S_{yx} }}{\partial x} + \frac{{\partial S_{yy} }}{\partial y}} \right) + \frac{\partial }{\partial x}\left( {hT_{xy} } \right) + \frac{\partial }{\partial y}\left( {hT_{yy} } \right) + hv_{s} S \hfill \\ \end{gathered} $$

where *t* is the time; *η* is the water level; *d* is the static water depth; *h* = *η* + *d* is the total water depth; the velocity components in the *x*, *y* direction of *u* and *v*, respectively; *f* is the Coriolis parameter; *S* is the source emissions; *S*_*xx*_, *Sxy*, *S*_*yx*_, and *S*_*yy*_ are the components of the radiant stress tensor, respectively. *τ*_*sx*_ and *τ*_*sv*_ are surface wind stresses, and *τ*_*bx*_ and *τ*_*bv*_ are bottom stresses. *T*_*xx*_, *T*_*xy*_, and *T*_*yy*_ are lateral stresses; *u*_*s*_, *v*_*s*_ are the flow velocity of the source and sink terms; the letter with a dash is the mean of that physical quantity.

#### Water quality model

We used the Transport Module (AD) to calculate the water quality model. Transport Module is a mathematical description of the pattern of change of pollutants in the water environment and the interrelationships between their influencing factors, which is both one of the contents of scientific research on the water environment and an important tool for water environment research^28^. In this paper, the Transport Module of the Mike21 was selected as the water quality module, and the transport and diffusion equations of pollutants are given in the following equation^29^:

4 $$ \frac{\partial }{\partial t}\left( {hC} \right) + \frac{\partial }{\partial x}\left( {uhC} \right) + \frac{\partial }{\partial y}\left( {vhC} \right) = \frac{\partial }{\partial x}\left( {hD_{x} \frac{\partial C}{{\partial x}}} \right) + \frac{\partial }{\partial y}\left( {hD_{y} \frac{\partial C}{{\partial y}}} \right) + F\left( C \right) + S $$

where *D*_*x*_ and *D*_*y*_ are the turbulent diffusion coefficients in the *x* and *y* directions, respectively. *C* is the composite concentration (constant); *F(C)* is the biochemical reflection term.

### The percentage bias coefficient

The Percentage Bias Coefficient (PBIAS) is frequently employed to assess the accuracy of water quality simulation outcomes from the model. This coefficient characterizes the extent of disparity between the measured and simulated values^30^. The formula for PBIAS is as follows^31^:

5 $$ PBIAS = \left| {\frac{{\sum\limits_{i = 1}^{n} {\left( {M_{v} - C_{v} } \right)} }}{{\sum\limits_{i = 1}^{n} {M_{v} } }}} \right| \times 100\% $$

where *M*_*v*_ represents the measured value, *C*_*v*_ stands for the simulated value, and *n* denotes the number of measured data points. Typically, in water quality simulation outcomes, PBIAS ≤ 25% signifies excellent results, 25% < PBIAS ≤ 40% indicates good results, while values beyond these ranges suggest fair results.

### Water quality evaluation

The single factor pollution index is often used as a method of evaluating individual pollution factors, and the measured value of the index is compared with the standard limit of the index in the *Environmental Quality Standard for Surface Water (GB 3838-2002)*, and the category of the worst index is used as the final water quality category. Should the indicator surpass the standard value associated with its respective functional category, it indicates that the water body fails to meet the pertinent water quality standards^32^. The method is simple and can help determine main pollutants and the relationship between the water quality status and assessment standards^33,34^. The calculation of the single factor index method can be expressed as^35^:

6 $$ SI = \left( {SI_{i} } \right)_{\max } = \left( {\frac{{C_{i} }}{{S_{i} }}} \right)_{\max } $$

where SI is the single-factor pollution index of the pollutants; C_i_ is actual concentration of pollutants i; S_i_ is the evaluation standard value of pollutant i; and (SI_i_)max is the maximum classification for the pollutant parameters (the most polluted parameter).

## Model construction and verification

### Model construction

#### Mesh processing

**Figure 2:**
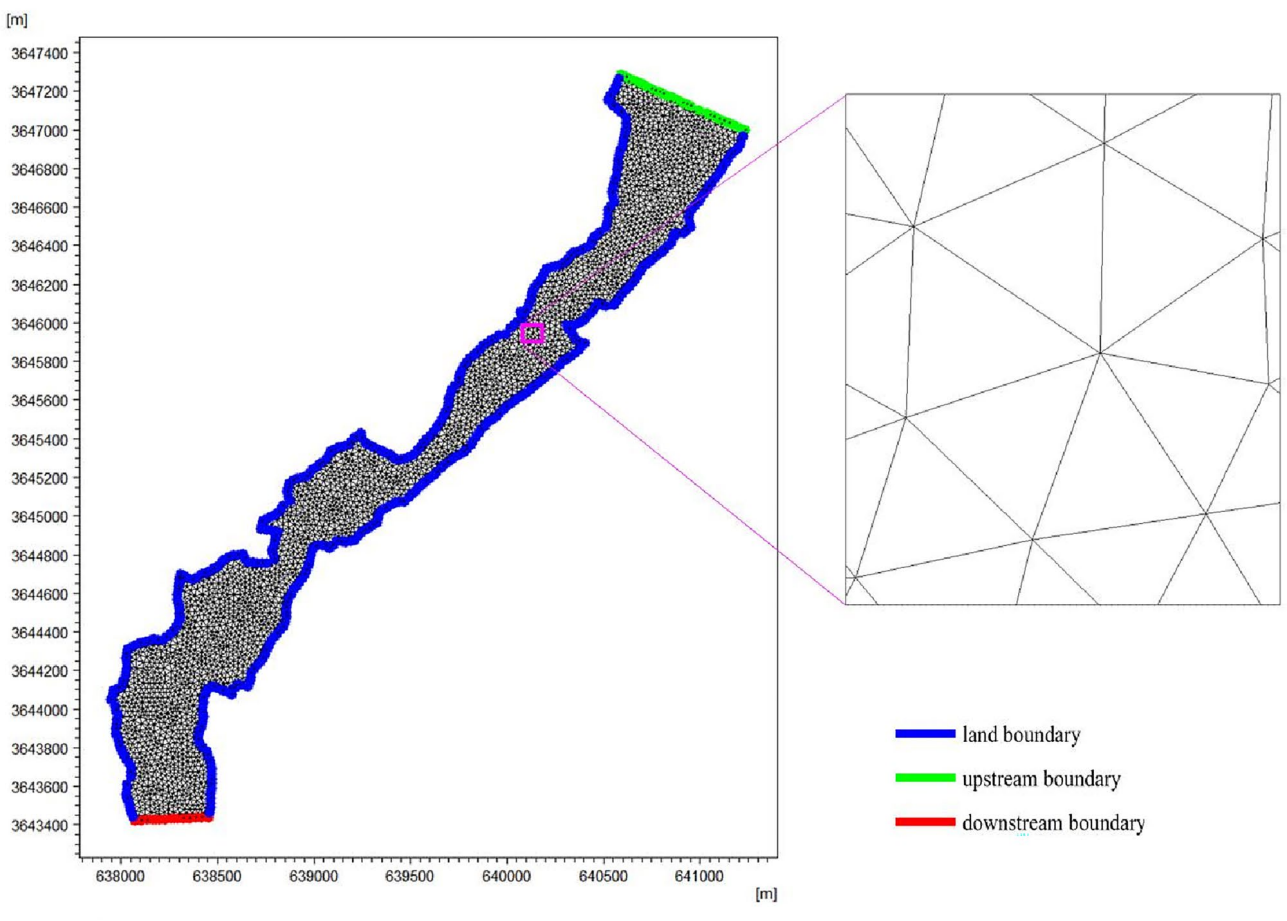
Grid division of the study area.

Since the grid generated by the SMS grid generator is more advantageous in terms of computational speed and convergence rate of the solution, this paper utilized the SMS for mapping the grid topography of the Bai River^36^. By utilizing the measured data, we can acquire the geographical coordinates of points along the Bai River, in the SMS to draw the land and water boundary lines and smooth processing, and then use the unstructured triangular mesh to generate the triangular mesh delineation map, and screen the grid, adjusted the minimum angle of less than 30° of the mesh to reach the standard manually^37^, the production of the mesh delineation map is shown in Fig. 2. The maximum cell side length of the grid in the grid topographic map is set to 50 m, the maximum cell area is set to 500 m^2^, and there are 5623 triangular adaptive grids. Finally, the topographic interpolation of the gridding map was carried out to generate the calculation grid, and the corresponding elevation data of the river came from the measured data of Nanyang Hydrology and Water Resources Survey Bureau.

#### Model boundary conditions

When setting the Mike21 model boundary conditions, it is generally accepted that the upstream boundary with inflow data and water level data as the downstream boundary condition could yield a more convergent and stable model^38^. The upstream boundary condition for model validation was the 2019 day-by-day inflow data at the Fourth Rubber Dam section of the Bai River, and the downstream boundary condition was the 2019 day-by-day outflow monitoring water level at the Shangfanying section of the Bai River.

#### Model parameter setting

In order to fit the natural geographical characteristics of the study river, so as to simulate the water environment condition, we chose CFL, Eddy viscosity, Manning number as the main parameters of the hydrodynamic model, and Diffusion coefficient, pollution factors degradation rate as the main parameters of the water quality model^39^. According to the water quality monitoring results of the assessment section of the Bai River in 2017 to 2021, COD, NH_3_-N, and BOD_5_ were selected as the water quality indicators for model. After calibrated by the measured data of the Bai River in 2018, the value of the model parameters were shown in the Table 1.

**Table 1:** Parameter settings of the model.

Parameter	Value
CFL	0.8
Eddy viscosity/(m^2^/s)	0.28
Manning number/(m^1/3^/s)	32
Diffusion coefficient/(m^2^/s)	0.658
COD degradation rate/(mg/d)	0.25
NH_3_-N nitrification rate/(mg/d)	0.12
BOD_5_ degradation rate/(mg/d)	0.4

### Model validation

#### Hydrodynamic model validation

**Figure 3:**
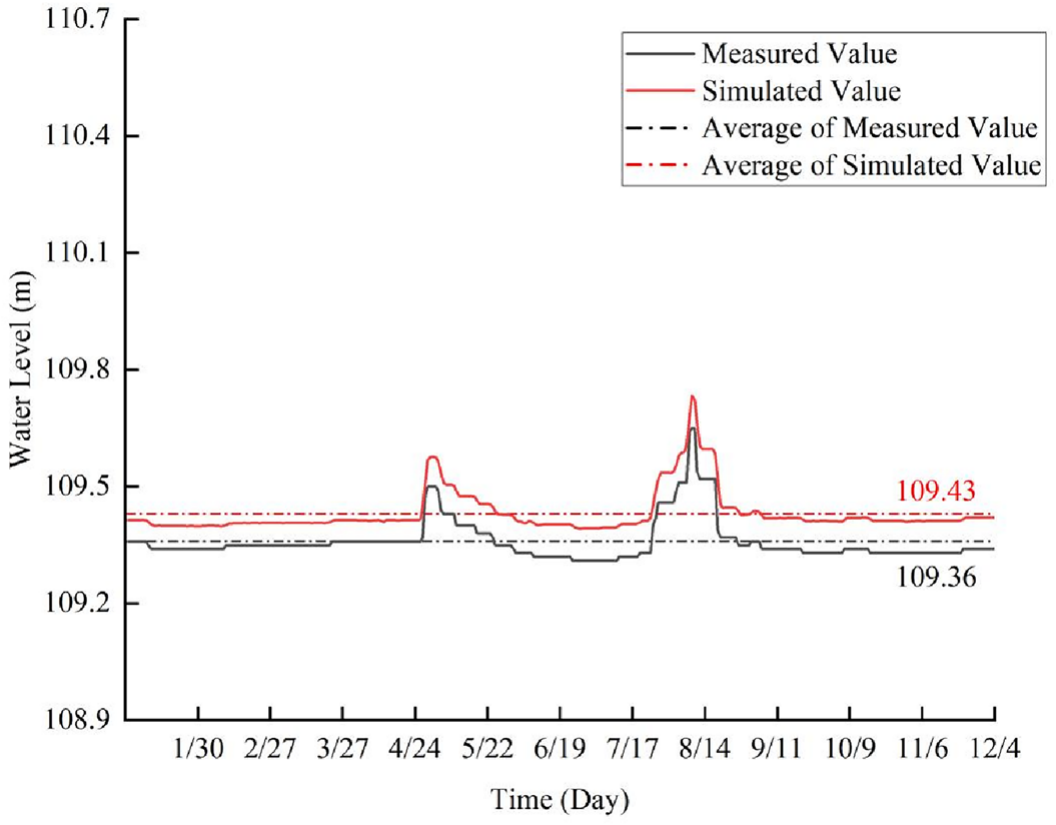
The validation results of the water level.

Day-by-day measured water level data of P1 from 1 January to 5 December 2019 were selected for hydrodynamic module validation, and the water level validation results are shown in Fig. 3. The simulated water level values and their change trends closely aligned with the measured data in the model. The maximum absolute error between simulated and measured water levels is 0.08 m, with a maximum relative error of 0.073%. These findings indicate the reasonableness of the simulation results^26^.

#### Water quality model validation

**Figure 4:**
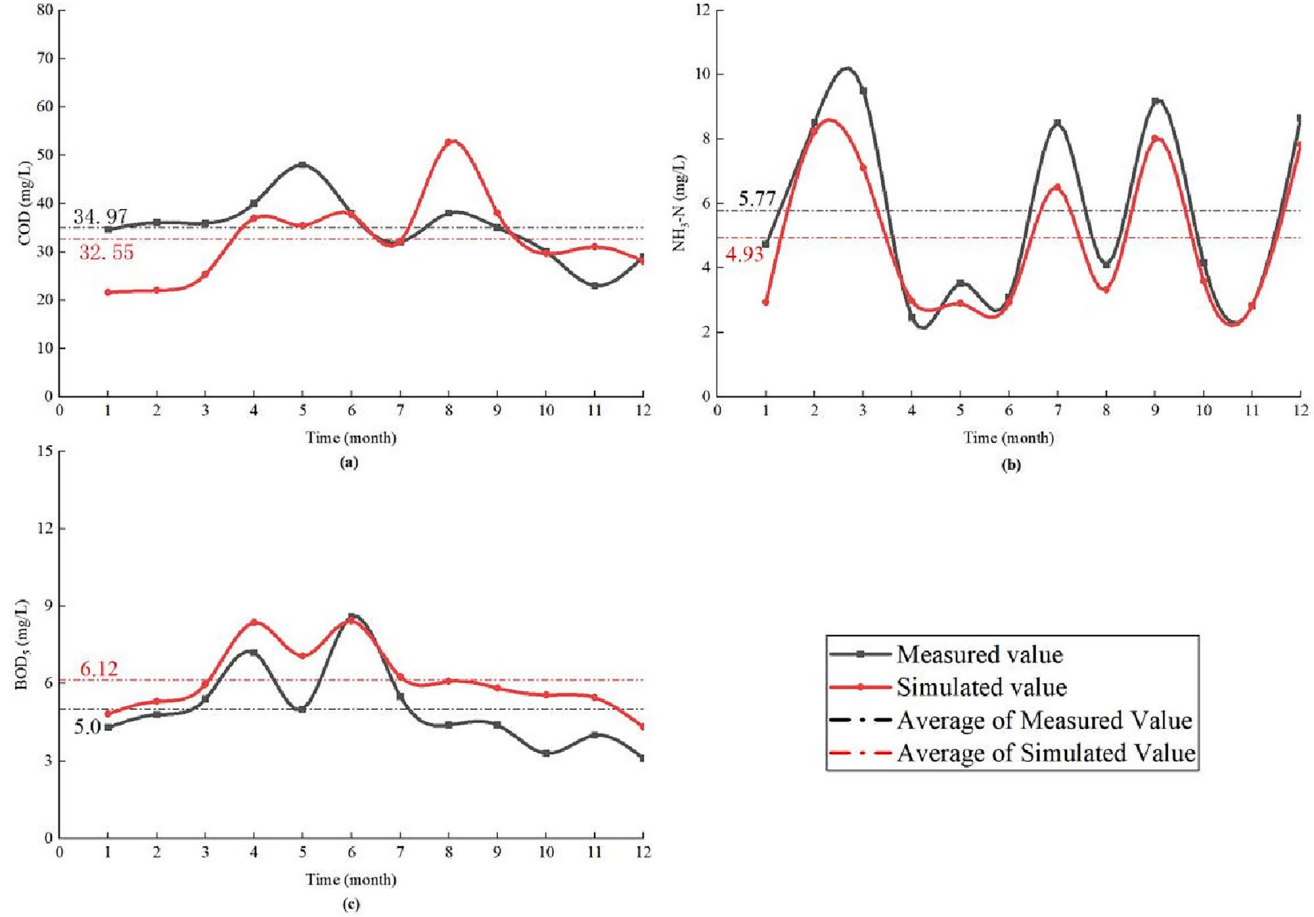
The validation results of the water quality. (**a**) COD, (**b**) NH_3_-N, (**c**) BOD_5_.

The validation of the water quality module centered on P2 as the validation point. Measured concentrations of COD, NH_3_-N, and BOD_5_ were selected for each month from January to December 2019 to compare with the corresponding simulated concentrations, and the water quality validation results were shown in Fig. 4. The PBIAS of COD, NH_3_-N and BOD_5_ in the simulation results of the water quality module are 19.3%, 16.2% and 23.1% respectively, which are all less than 25%, indicating that the simulation results were excellent, and it can be used for this study.

## Results and discussion

### Scenario setting

Based on the actual situation of the construction of NSPC, the project were divided into three working conditions, from the operation stage of the first and second phases of NSPC in 2019 to the operation of the third phase of NSPC, and used the SMS-Mike21 coupling model to explore the impact of the three working conditions on the water environment under different hydrology periods. It should be added that we selected three water quality indicators: COD, NH_3_-N, and BOD_5_, and assumed that the concentration of water quality indicators of sewage discharged from each sewage discharge outlet will remain unchanged after the expansion of NSPC, and only the amount of sewage discharge will be changed. The working conditions are set in Table 2.

**Table 2:** Pollutant discharge scenario setting.

Hydrology period	working condition	Water inflow from the upstream boundary/(m^3^/s)	Quality concentration of upstream boundary/(mg/L)			Sewage discharge OF the sewage outlet/(10^4^m^3^/d)
			COD	NH_3_-N	BOD_5_	
The wet season	CI	41.3	25.2	0.27	3.04	S1 = 5, S2 = 20, S3 = 8
	C2					S1 = 0, S2 = 25, S3 = 8
	C3					S1 = 0, S2 = 40, S3 = 8
The normal season	CI	5.05	25.5	0.34	3.93	S1 = 5, S2 = 20, S3 = 8
	C2					S1 = 0, S2 = 25, S3 = 8
	C3					S1 = 0, S2 = 40, S3 = 8
The dry season	CI	1.79	27	0.18	3.9	S1 = 5, S2 = 20, S3 = 8
	C2					S1 = 0, S2 = 25, S3 = 8
	C3					S1 = 0, S2 = 40, S3 = 8

Condition 1 (C1): In 2019, the first and second phase of NSPC was in operation stage. NSPC discharged 50,000 m^3^/d untreated sewage from S1 and 200,000 m^3^/d treated sewage from S2; meanwhile, NTWTP discharged 80,000 m^3^/d treated sewage from S3 during this period.

Condition 2 (C2): The future third phase of NSPC has been completed but is not running at full capacity. NSPC did not discharge sewage from S1, and discharging 250,000 m^3^/d treated wastewater from S2, S3 of NTWTP emissions remain unchanged.

Condition 3 (C3): Full-load operation of the third phase of NSPC. NSPC did not discharge sewage from S1, discharged treated sewage 400,000 m^3^/d from S2. Under the condition, NTWTP kept the unchanged discharge from S3.

To sum up, in terms of the amount of water inflow in different hydrology periods, the maximum upstream water inflow in the wet season in 2019 was 41.3 m^3^/d. In the dry season, the minimum upstream water flow was 1.79 m^3^/d. In terms of the water quality of different hydrology periods, the difference between the upstream water quality concentration of the three hydrology periods was small, and the COD concentration was the largest in the dry season, which was 27 mg/L. The concentrations of NH_3_-N and BOD_5_ were the highest in the normal season, which were 0.34 mg/L and 3.93 mg/L, respectively. This paper explored the impacts of three working conditions on the water environment in different hydrology periods, which is conducive to the government's better response to the impacts of Nanyang City's wastewater discharge on the Bai River in different stages of development, and to provide effective measures for the management of the water environment.

### Water quality evaluation results

**Figure 5:**
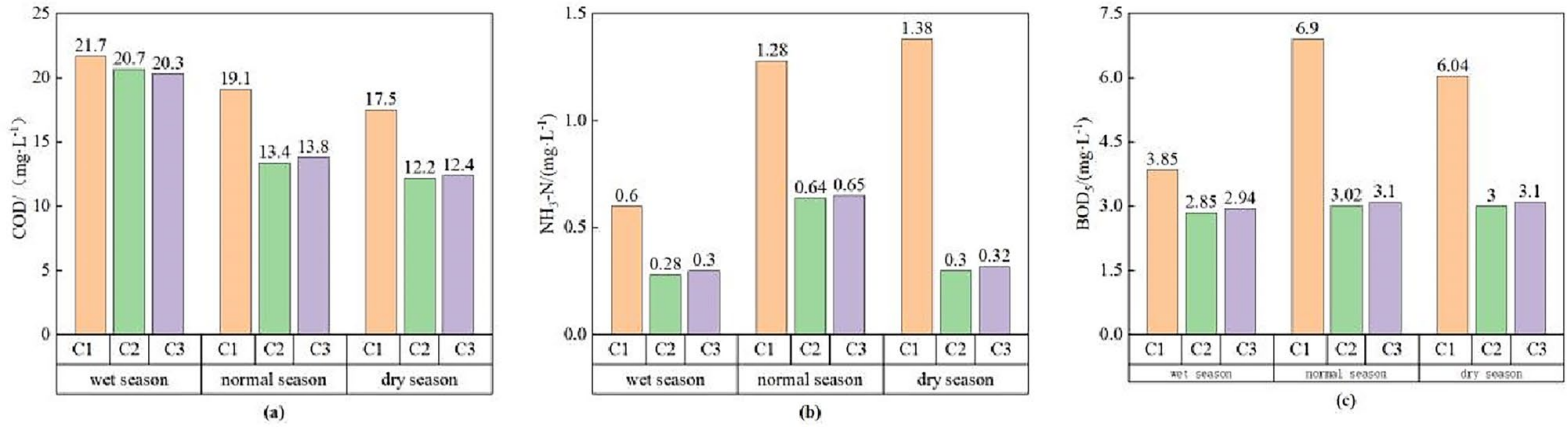
Simulation value of the pollution factor concentration in the assessment section under different sewage discharge scenarios. (**a**) COD, (**b**) NH_3_-N, (**c**) BOD_5_.

In this study, the SMS-Mike21 hydrodynamic-water quality coupling model was employed to simulate variations in COD, NH_3_-N, and BOD_5_ concentrations at the assessment section under three distinct operational conditions during different hydrology periods, and the simulation results were showed in Fig. 5. Based on Fig. 5, we used the Single-factor evaluation method and single-factor pollution index to evaluate the simulation results of each indicator, and the evaluation results are shown in the Table 3. In the past, when considering different hydrological periods, water quantity was considered often and water quality was ignored, which would lead to ignoring the influence of incoming water quality on the water environment simulation results and not obtaining sufficient accurate research results^24,25^. In this paper, the differences in water quality between different hydrological seasons were considered to make the simulation more realistic.

**Table 3:** Assessment results of the water quality in the assessment section of the Bai River.

Hydrology period	working condition	COD	NH_3_-N	BOD_5_	SI	Assessment results
The wet season	CI	IV (1.085)	III	III	1.085	IV
	C2	IV (1.035)	II	I	1.035	IV
	C3	IV (1.015)	II	I	1.015	IV
The normal season	CI	III	IV	V (1.725)	1.725	V
	C2	I	III	III (0.755)	0.755	III
	C3	I	III	III (0.775)	0.775	III
The dry season	CI	III	IV	V (1.51)	1.51	V
	C2	I	II	II (0.75)	0.75	II
	C3	I	II	III (0.775)	0.775	III

Under C1, the concentration of the three indicators of the assessment section in each hydrological period is higher than the concentration under C2 and C3, because the most pollutants entered the river under C1. The water quality category evaluation results of assessment section in each hydrological period were shown in Table 3, it can be seen that the water quality evaluation results in the wet season, normal season and dry season were class IV, V and V respectively, and the water quality category was the best in the wet season, which was due to the stronger dilution effect of the largest upstream water volume on the river pollutants. Besides, the most polluted parameter (SI) of the wet, normal and dry season were COD (1.085), BOD_5_ (1.725) and BOD_5_ (1.51) respectively. Therefore, under C1, the government needs to pay attention to the treatment of COD in the wet season, and the treatment of BOD_5_ in the normal and dry season.

Under C2, the concentrations of the three indicators in each hydrological period of the assessment section decreased to varying degrees compared with C1, because the discharge of untreated sewage from S1 was stopped under C2, and the pollutants entering the river were reduced. The water quality category evaluation results of assessment section in each hydrological period were shown in Table 3, it can be seen that the water quality evaluation results in the wet season, normal season and dry season were class IV, III and II respectively, among which the water quality category in the dry season was upgraded from class V to class II, and the improvement effect is the most obvious, followed by the water quality category in the normal season was upgraded from class V to class III, and the water quality category in the wet season was class IV, which had not changed, because the C2 was to discharge the treated water into the Bai river, diluting the polluted river. The most obvious reason for the dilution effect in the dry season was that the amount of water coming from the upstream is the least, and the dilution pressure is the lowest during the dry season. It can be seen from Table 3, in the wet season, only the COD concentration exceeded the class III water quality standard, which is the excess multiple of 1.035, showing a slight decrease compared to the COD concentration in C1. Under C2, no pollution factor concentration exceeded the class III water quality standard in the normal and dry season. Compared with C1, the BOD_5_ concentration exceeds the standard, it can be seen that the shutdown of S1 had a great positive effect on the pollution prevention and control of BOD_5_, and it can be inferred that the amount of BOD_5_ in the untreated sewage in the urban area of Nanyang is higher.

Under C3, the water quality evaluation results in the wet season, normal season and dry season were class IV, III and III, respectively, In the wet season, only the COD concentration exceeded the water quality standard of Class III, which was Class IV, and the excess multiple was 1.015, but there was a slight decrease in the COD concentration relative to C2. In the normal season and the dry season, no pollution indicactor’s concentration exceeded the water quality standard of class III. Compared with C2, the water quality category of C3 did not change in the wet season and normal season, and only the water quality category was reduced from class II to III in the dry season, which was because the concentration simulation value of BOD_5_ changed from class II to III. It can be speculated that after the full load operation of the third phase of NSPC, because the purification of BOD_5_ by NSPC is not complete, with the increase of sewage discharge, the amount of water inflow decreases, and the concentration of BOD_5_ in the assessment section increases. The government needs to increase the treatment of COD in the wet season and BOD_5_ in the dry season, and at the same time, the NSPC needs to upgrade the purification technology and purification equipment of BOD_5_. Hence, following the full-load operation of NSPC’s third phase, incomplete BOD_5_ purification by NSPC, coupled with rising sewage discharge and decreasing upstream water inflow, leads to BOD_5_ concentration increased in the assessment section. Addressing this, the government should intensify COD treatment during the wet season and BOD_5_ treatment in the dry season. Simultaneously, NSPC needs to upgrade BOD_5_ purification technology and equipment for enhanced efficiency.

In conclusion, the expansion of the NSPC improved the water quality category of the assessment section. In C1, the water quality category of the assessment section was the worst, the water quality category of C2 and C3 had been greatly improved compared with C1. the main reason was the shut down of untreated sewage outlet S1. In C3, at full capacity, the increase of NSPC discharge led to no change in the water quality category of the Bai River during the wet season and normal season compared to C2. However, there was a slight increase in BOD_5_ concentration during the dry season, resulting in a marginal decline in the overall water quality of the Bai River. It can be seen that the expansion of NSPC had improved water quality efficiently^24,25^, but the purification capability of BOD_5_ is weak, and the purification technology needs to be further improved. This provided scientific guidance for the formulation of the Bai River’ water environment problems in the future.

**Figure 6:**
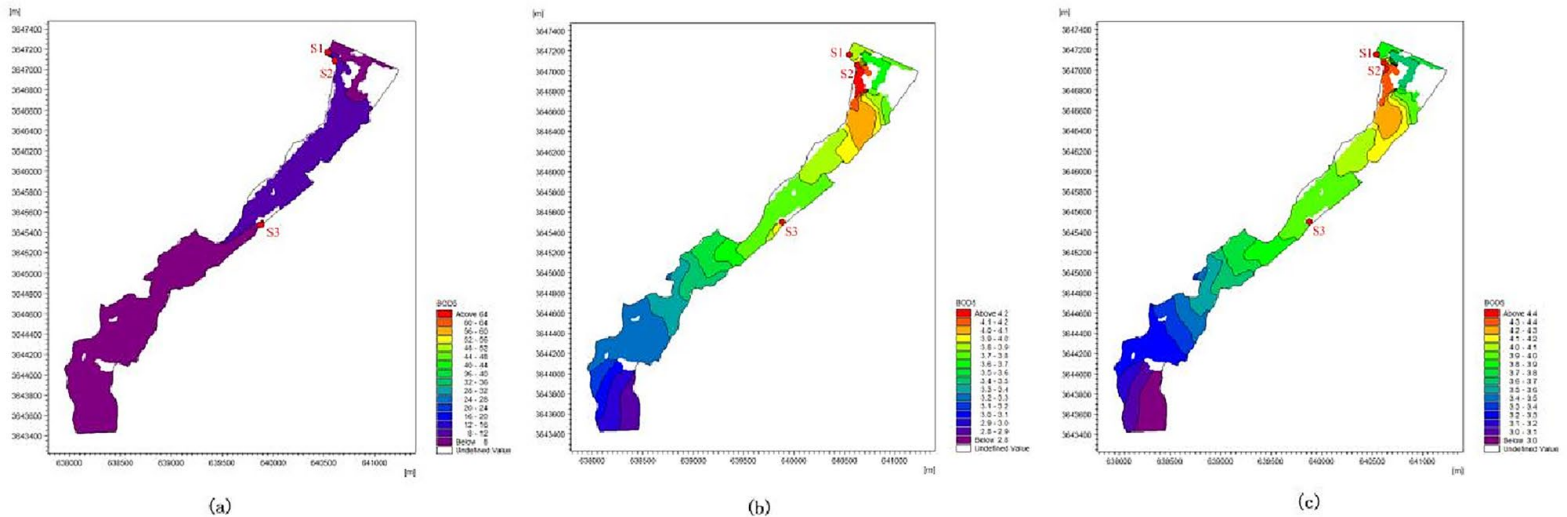
Spatial variation characteristics of BOD_5_ concentration under three conditions during the dry season (January, February, and December): (**a**) C1, (**b**) C2, (**c**) C3.

According to the above analysis results, from the operation stage of the first and second phases of NSPC in 2019 to the completion of the construction of the third phase in the future, the BOD_5_ concentration in sewage significantly influences the water quality category of the assessment section of the Bai River. Although the simulated value of water quality concentration varied in different hydrological periods, the spatial variation of concentration is basically the same. Therefore, we only chose the dry season as the hydrological background to explore the spatial variation characteristics of BOD_5_ concentration under the three working conditions during the dry season, as shown in the Fig. 6.

Spatially, the concentration of BOD_5_ decreased significantly from C1 to C2 during the dry season, but increased slightly from C2 to C3. Under C1, the overall BOD_5_ concentration value of the river is too high, and the concentration variation range from 6 to 65 mg/L, which exceeded the class III surface water quality standard of BOD_5_ (4 mg/L), the area with elevated pollution levels is situated in the upper reaches of the river, where the overall BOD_5_ concentration exceeds 8 mg/L. This is primarily attributed to the presence of sewage outlets S1 and S2 in the upper reaches, with S1 discharging untreated sewage directly, leading to severely degraded water quality and significantly exacerbating the pollution situation in the Bai River section^40,41^. Under C2, the overall concentration value of BOD_5_ in the river is lower than that of C1, and the variation range of concentration is 3–4.3 mg/L, and the pollution high value area of BOD_5_ was located near the sewage outlet S2, and the concentration value exceeded the quality standard of class III surface water, which is due to the large discharge of S2 sewage outlet compared with S1, and the purification capability of NSPC for BOD_5_ is weak, resulting in the pollution high value area of BOD_5_ near S2. As the pollutants moved away from S2, the BOD_5_ in the river channel gradually decreased due to the self-purification capability of the river. However, the closer to S3, the concentration of BOD_5_ gradually increased, and then when the pollutant diffused to the downstream of the river, the dilution effect of the upstream to the downstream led to the gradual decrease of the pollutant concentration. In C3, the overall concentration value of BOD_5_ in the river was slightly higher than that in C2, and the variation range of BOD_5_ concentration was 3.1–4.5 mg/L. BOD_5_ near S2 reached the highest value, which exceeded the Class III surface water quality standard. The spatial distribution of BOD_5_ concentrations was the same as in C2.

In summary, from the spatial point of view, the overall spatial distribution of BOD_5_ concentration under the three working conditions in the dry season showed that the downstream was better than the upstream, which was caused by the dilution from upstream to downstream.

Based on the above analysis, it was found that the BOD_5_ concentration of C3 in the dry season was slightly worse than C2, due to the sharp increase in sewage discharge and the insufficient purification capability of BOD_5_ by the NSPC. Therefore, BOD_5_ is the main pollutant discharged from outlet and the most important factor leading to the deterioration of river water quality, so it is suggested that NSPC should actively fetch in new sewage purification technologies to further improve the sewage purification effect in the future, so as to reduce the concentration of pollution factors in the treated sewage, and improve the removal rate of BOD_5_^23^.

Physical, chemical, and biological remediation techniques can be used to improve BOD_5_ removal rate^42^. Physical remediation technology: the main principle is to remove pollutants in the river channel by means of dredging sediment, mechanical algae removal, water diversion, dredging and water transfer, etc., in this way to enhance the hydrodynamic conditions of the river, so as to improve the fluidity of the water body and enhance the dilution and degradation ability of pollutants^43,44^. Chemical remediation technology: the main principle is to use chemical agents that can react with specific pollutants to coagulate and precipitate pollutants or eliminate algae, and reduce the concentration of pollutants in the river. Bioremediation technology: the main principle is to use the metabolic activities of specific organisms (plants, microorganisms or protozoa) to absorb, transform, eliminate or degrade environmental pollutants to achieve environmental purification and ecological effect restoration^45^. In the actual process of water environment restoration, one kind of technology or a combination of technologies can be selected in combination with the actual situation of the restoration object, so as to achieve the purpose of water environment restoration better and faster^46^. This study pointed out the key treatment points and improvement ideas for the improvement of the water quality category and the overall water environment status of the main sewage discharge area of the Bai River (Nanyang Section).

## Conclusion

In this paper, we considered the variation of water quantity and water quality in different hydrological periods, and established a two-dimensional hydrodynamic-water quality coupling model by SMS and Mike21 to make the model simulation results more available. This approach allows for a more comprehensive study into the impact of NSPC expansion on the water environment in the main sewage discharge area of the Bai River (Nanyang section). Therefore, SMS-Mike21 coupling model has good accuracy and applicability for simulating and predicting water environments in similar scenarios.

After the expansion of NSPC, the untreated sewage outlet S1 was closed, and the water quality of the Bai River was significantly improved in the normal season and the dry season, among which the water quality in the dry season was the most obvious. However, with the increase of NSPC sewage discharge, the concentration of BOD_5_ in the assessment section increased slightly in the dry season, leading to a slight decrease in the water quality category of the section. Hence, while the expansion of NSPC contributes to enhancing water quality, the weak purification capability for BOD5 highlights the need for further improvements in purification technology.

From a spatial point of view, the water quality in the upper reaches of the river is superior to that in the lower reaches, attributed to the dilution effect of the water. Besides, due to significant pollutant discharges, localized increases in pollutant concentrations were observed near the three outlets^47^.

BOD_5_ is the primary factor contributing to the degradation of water quality in the river assessment section, the impact of BOD_5_ concentration on the overall water quality category of the river is particularly evident during the dry season. For a large amount of BOD_5_ in the Bai River (Nanyang section), we recommend the remediation of BOD_5_ by physical, chemical, and biological methods^41–43^.

This study provided SMS-Mike21 coupling model for the water environment simulation, pointed out the water environment simulation in different periods should pay attention to the difference in water quality. It offered targeted insights for improving river water quality, laying a scientific foundation for urban water environment control. Additionally, it provides guiding recommendations for future studies on water environment condition simulations. Future studies can enhance and broaden the model by include non-point source contamination into the model, thus rendering the water environment model more comprehensive.

## Data Availability

The data that support the finding of this study are available from the corresponding author upon reasonable request.
